# Rivaroxaban-Induced Nontraumatic Spinal Subdural Hematoma: An Uncommon Yet Life-Threatening Complication

**DOI:** 10.1155/2015/275380

**Published:** 2015-10-12

**Authors:** Mazen Zaarour, Samer Hassan, Nishitha Thumallapally, Qun Dai

**Affiliations:** ^1^Department of Medicine, Staten Island University Hospital, North Shore-LIJ Health System, Staten Island, NY 10305, USA; ^2^Department of Medicine, Division of Hematology and Oncology, Staten Island University Hospital, North Shore-LIJ Health System, Staten Island, NY 10305, USA

## Abstract

In the last decade, the desire for safer oral anticoagulants (OACs) led to the emergence of newer drugs. Available clinical trials demonstrated a lower risk of OACs-associated life-threatening bleeding events, including intracranial hemorrhage, compared to warfarin. Nontraumatic spinal hematoma is an uncommon yet life-threatening neurosurgical emergency that can be associated with the use of these agents. Rivaroxaban, one of the newly approved OACs, is a direct factor Xa inhibitor. To the best of our knowledge, to date, only two published cases report the incidence of rivaroxaban-induced nontraumatic spinal subdural hematoma (SSDH). Our case is the third one described and the first one to involve the cervicothoracic spine.

## 1. Introduction

Historically, warfarin, a vitamin K antagonist (VKA), was the only available oral anticoagulant (OAC). In the last decade, the need for new OACs emerged, as warfarin has multiple food and drug interactions and requires frequent monitoring [1]. Rivaroxaban is a factor Xa inhibitor with numerous characteristics allowing this drug to be an attractive alternative to VKA. In addition to noninferiority compared to warfarin in selected clinical scenarios, rivaroxaban has the advantage of once daily dosing, faster onset of action, larger therapeutic index obviating the need for close monitoring, and lower risk of bleeding [2, 3]. However, although the rate of rivaroxaban-associated bleeding is reduced, the risk still remains.

Intracranial hemorrhage (ICH) is one of the most common bleeding complications associated with OACs. Traumatic spinal hematoma is a well-known complication following the administration of these agents. On the other hand, nontraumatic spinal hematoma is a relatively uncommon yet life-threatening neurosurgical entity. To our knowledge, only two cases in the literature describe nontraumatic spinal subdural hematoma (SSDH) in the setting of rivaroxaban therapy (Table 1). Our case is the third case reported and the first one to involve the cervicothoracic spine. Informed consent was obtained from the patient prior to publication.

## 2. Case Presentation

We report the case of a 58-year-old male that presented to our Emergency Department with complaints of acute interscapular back pain and bilateral lower extremities weakness.

Patient's medical history was significant for diabetes mellitus type II, hypertension, dyslipidemia, and atrial fibrillation. His surgical history consisted of umbilical hernia repair and left hip arthroplasty. Patient had no allergies and had stopped smoking many years ago. He was diagnosed with atrial fibrillation 30 days prior to admission, for which he was started on rivaroxaban 20 mg once daily. The rest of his medications included atorvastatin, fish oil, sitagliptin, quinapril, and triamterene-hydrochlorothiazide.

On the day of admission, the patient admitted waking up at 3:00 am with sudden onset, sharp, interscapular back pain. He denied any similar pain in the past. The patient first presented to another institution, where he started having bilateral lower extremities numbness, which progressed rapidly to bilateral weakness. At this point, he was still able to walk but with difficulty. He was then transferred to our institution, where his weakness became more pronounced. Patient denied any other neurologic symptoms. There was no history of trauma. Prior to this, he had a left hip arthroplasty three weeks earlier, which was performed under spinal (lumbar) anesthesia, without complications. Rivaroxaban was stopped three days before the surgery. Postoperatively he did not receive nonsteroidal anti-inflammatory drugs and was restarted on the OAC few days later. Moreover, he reported driving for almost 7 hours from Elmira (New York) to Staten Island (New York) one day prior to presentation.

Initial physical examination revealed an alert and oriented patient in no acute distress, with a Glasgow Coma Scale score of 15. His blood pressure was 148/98 mmHg; heart rate was 82 beats/min and irregular; body temperature was 98.7°F; respiratory rate was 18 breaths/min. Cardiopulmonary and abdominal exams were within normal limits. Tenderness was elicited over the upper thoracic spine. There were no neurological deficits in the cranial nerves or upper limbs. The motor strength in both lower limbs was of Medical Research Council grade 0/5. A sensory level was evident at the level of T2 with positive bilateral Babinski reflexes.

Initial laboratory assays included a hemoglobin concentration of 11 g/dL, a platelet count of 176 × 10^9^/L, an activated partial thromboplastin time (aPTT) of 36.6 seconds, a prothrombin time (PT) of 18.4 seconds, and an international normalized ratio (INR) of 1.6. Kidney function and liver enzymes were normal. Electrocardiogram and chest radiograph were unremarkable. A noncontrast computerized tomography (CT) of the brain was negative for bleed, and a CT of the chest was also negative for dissection.

**Figure 1:**
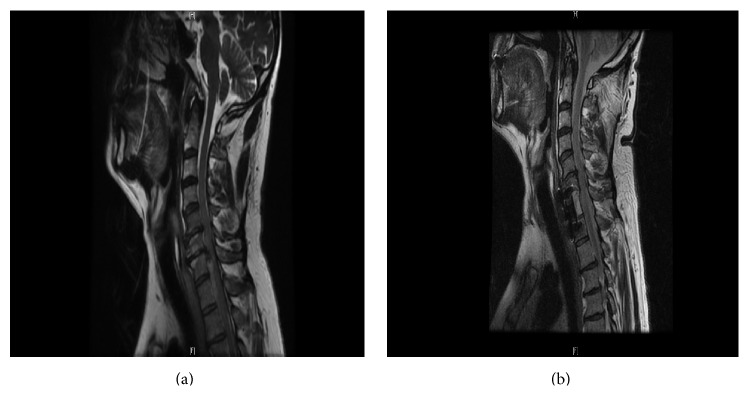
(a) T2-weighted sagittal image of the cervicothoracic spine illustrates a hyperintense 6.3 × 0.6 × 1.6 cm fluid collection from C7 to T2 vertebral bodies, with complete effacement of the CSF space and spinal cord edema. (b) T2-weighted sagittal image of the cervicothoracic spine performed 6 weeks after the surgical intervention shows resolution of the spinal fluid collection.

The clinical presentation combined with findings on the neurological exam pointed towards a spinal pathology. Moreover, a spinal bleed was strongly suspected given the history of recent spinal anesthesia in addition to the rivaroxaban use. An emergent cervical and thoracic magnetic resonance imaging (MRI) demonstrated an acute 6.3 × 0.6 × 1.6 cm intradural hematoma from C7 to T2, with spinal cord edema (Figure 1(a)). Neurosurgical intervention was not deemed an option at this point given that the increased risk of rivaroxaban-induced bleeding outweighed the benefits. Therefore, a conservative management was adopted, including close neurological monitoring in the intensive care unit along with high dose intravenous dexamethasone (100 mg once followed by 10 mg every 6 hrs). Patient was also offered prothrombin complex concentrate (PCC), but he declined to proceed with this therapeutic option given its potential side effects, such as prothrombotic state, as well as its uncertain efficacy in similar spinal bleed. He was started instead on aminocaproic acid.

The following day, patient's neurological exam had improved. Sensory level was down to T4-T5, and motor strength was 4/5 in the right lower extremity while it remained 1/5 on the left lower extremity. Given the increased risk of bleeding, especially taking into account the minor improvement in the patient's condition, surgical intervention was delayed for three days, to allow complete elimination of rivaroxaban. On hospital day 4, patient underwent a successful and uneventful anterior C7 corpectomy with resection of a large intradural hematoma. Postoperatively, his neurological exam continued to improve slowly, and he was discharged for intensive rehabilitation 5 days after the surgery. A repeat MRI done 6 weeks later showed almost complete resolution of the spinal hemorrhage (Figure 1(b)).

## 3. Discussion

For over 50 years, the only available OAC was warfarin. Although it has proven efficacy, a number of limitations make its use challenging for physicians, including numerous food and drug interactions, delayed onset of action, and narrow therapeutic index necessitating continuous INR monitoring [1]. Last decade has seen an explosion of target-specific OACs, which directly inhibit either factor Xa or thrombin. Rivaroxaban has emerged as one of four current alternatives to warfarin, along with dabigatran, apixaban, and edoxaban.

Rivaroxaban (Xarelto), an oral oxazolidinone-based anticoagulant, is a potent direct factor Xa inhibitor. The Food and Drug Administration (FDA) approved rivaroxaban for the prevention of venous thromboembolism (VTE) in patients undergoing knee or hip replacement surgeries, secondary prevention of recurrent VTE, treatment of deep vein thrombosis and pulmonary embolism, and the reduction of stroke risk in patients with nonvalvular atrial fibrillation [2, 3, 9].

Rivaroxaban is a substrate of CYP3A4/5, CYP2J2, and the permeability glycoprotein (P-gp). Inhibitors and inducers of these CYP450 enzymes or transporters can lead to changes in rivaroxaban exposure. Therefore, the concomitant use of rivaroxaban with combined P-gp and strong CYP3A4 inhibitors (or inducers) should be avoided [2]. In our case, a drug-drug interaction with rivaroxaban seems unlikely, since the patient was not taking any strong cytochrome inducer/inhibitor.

In terms of toxicity profile, rivaroxaban was found to be associated with lower rates of major bleeding events when compared with warfarin in selected studies (Table 2). In fact, in the ROCKET AF trial [3], while gastrointestinal hemorrhages were more common in the rivaroxaban group compared to the warfarin group (3.2% versus 2.2% per year), the rates of ICH, the more devastating bleeding complication, were lower in the rivaroxaban group (0.5% versus 0.7% per year, hazard ratio HR = 0.67, *p* = 0.02). Moreover, in the EINSTEIN-PE trial [8], the risk of bleeding was found to be significantly lower with rivaroxaban compared to warfarin (HR = 0.49). Again, the major driver of fatal bleeding was ICH, which appears to be lower with rivaroxaban (RR = 0.48).

Spinal hematoma is an uncommon yet life-threatening neurosurgical entity. Depending on its location, the bleeding can be classified as epidural, intradural (subdural), subarachnoid, or intramedullary [10]. Nontraumatic or spontaneous spinal hematomas are rare and are usually epidural in origin [10]. Spontaneous SSDH are extremely rare and are associated with numerous risk factors, such as iatrogenic causes (lumbar puncture, spinal anesthesia, and spinal surgery), bleeding disorders, spinal tumors, arteriovenous malformations, and OACs [10–13].

The use of OACs is an uncommon risk factor for nontraumatic hematoma of the spine [12, 14]. We found only two cases of nontraumatic spinal hematoma associated with dabigatran therapy [15, 16]. Similarly, the incidence of such complication in the setting of rivaroxaban is unknown and is also only limited to case reports. To the best of our knowledge, to date, only four cases describe this dramatic entity (Table 1). While in the first two cases [4, 5] a rivaroxaban-associated nontraumatic epidural hematoma was highlighted, a SSDH was reported in the other two cases. Castillo et al. [6] describe a patient that developed a nontraumatic thoracolumbar SSDH extending from T3 to the conus medullaris, whereas Dargazanli et al. [7] report a patient that developed a thoracic nontraumatic SSDH. Our case is the third published case of rivaroxaban-induced SSDH and the first one to involve the cervicothoracic spine.

The exact underlying mechanism leading to spontaneous SSDH remains controversial, partly due to the rarity of this diagnosis. It is postulated that sudden increases of pressure in the thoracic and/or abdominal cavities can raise the pressure inside the subdural vessels. This leads to rupture of these vessels and subsequent SSDH [17]. In the current case, the reason for the SSDH may have been the combination of sustained increase in abdominal pressure (secondary to seat-belt use for more than 6 hours) and anticoagulation therapy. The contribution of the spinal anesthesia remains unclear, since it was performed in the lumbar spine, whereas the bleed occurred 21 days later in the cervicothoracic spine. This association between rivaroxaban-induced nontraumatic spinal bleed and the spinal anesthesia seems more obvious in the two reported cases of epidural hematoma in terms of timing and/or location of the bleed (Table 1).

In this context, the prescribing information for rivaroxaban carries a warning that patients who are anticoagulated and are receiving neuraxial anesthesia or undergoing spinal puncture are at risk for spinal or epidural hematomas [2]. Factors that can increase the risk of spinal hematomas in these patients include (1) use of indwelling epidural catheters, (2) concomitant use of other drugs that affect hemostasis, such as nonsteroidal anti-inflammatory drugs (NSAIDs), platelet inhibitors, and other anticoagulants, (3) history of traumatic or repeated epidural or spinal punctures, and (4) history of spinal deformity or spinal surgery.

The management of OAC-induced SSDH remains obscure. The lack of evidence-based guidelines, reversal strategy and specific antidote, makes physicians use nonspecific agents empirically. The management of VKA-associated spinal hematoma seems more straightforward with rapid reversal using fresh frozen plasma (FFP) and vitamin K, with or without PCC, followed by emergent surgical evacuation. In the setting of rivaroxaban, and in addition to discontinuing the drug, the use of agents such as PCC, activated prothrombin complex concentrate (aPCC), or recombinant factor VIIa may be considered but has not been evaluated in clinical trials [18, 19]. Because of the high plasma protein binding, rivaroxaban cannot be removed by dialysis. Moreover, if surgery is indicated, rivaroxaban should be discontinued at least 48 to 72 hours prior to procedure.

PCC and aPCC have been used for the most serious cases of OAC-associated bleeding. Several studies evaluated PCC, aPCC, and factor VIIa in animal bleeding models and in vitro, showing efficacy of these three products against factor X inhibitors [20, 21]. However, to date, there are no validated randomized trials to confirm these results. An extensive literature review on PubMed yielded one case where factor eight inhibitor bypassing activity (FEIBA) was used in a patient with rivaroxaban-associated subdural hematoma [22]. Taking into consideration the uncertain efficacy and the potential side effects of PCC, patients should be informed clearly about the rationale behind using these drugs and the possible adverse events [23].

Aside from correcting the coagulopathy, there is an ongoing debate regarding surgical intervention versus conservative management. The traditional standard-of-care treatment has been decompressive laminectomy and hematoma evacuation. Conservative management has been considered in cases with mild neurologic deficits, early spontaneous recovery, or high surgical risk patient [11].

## 4. Conclusion

Recently, the indications for OACs, including rivaroxaban, have expanded, and thus physicians should be aware of the increase in the incidence of spinal hematomas in patients receiving anticoagulant therapy. Available clinical trials showed lower incidence of rivaroxaban-associated major bleeding events, including intraspinal hemorrhage, compared to warfarin. However, even a single serious bleeding event secondary to OAC therapy is a devastating complication for the caregiver and for the patient. Therefore, establishment of a treatment algorithm or discovery of an antidote is of utmost importance.

## Figures and Tables

**Table 1:** Summary of the cases of rivaroxaban-associated nontraumatic spinal hematoma.

	Location and type of the spinal hematoma	Rivaroxaban dosage	Indication for rivaroxaban	Related spinal procedures	Management	Outcome
Jaeger et al. [4]	Cervicothoracic Epidural	10 mg daily	Postoperative prophylaxis	2 days after general anesthesia for proximal tibial osteotomy	Bed rest No surgery	Complete recovery

Radcliff et al. [5]	Lumbar Epidural	Unknown	Postoperative prophylaxis	7 days after spinal anesthesia for knee arthroplasty	Surgery	Complete neurologic recovery

Castillo et al. [6]	Thoracolumbar Subdural	20 mg daily	Atrial fibrillation	None	Cervical and lumbar drainage procedures	No recovery of bladder, bowel, and neurologic functions at 6 months after drainage

Dargazanli et al. [7]	Thoracic Subdural	20 mg daily	Atrial fibrillation	None	Prothrombin complex concentrate Surgery the next day	No improvement up to 6 months of follow-up

Zaarour et al. (our case)	Cervicothoracic Subdural	20 mg daily	Atrial fibrillation	21 days after lumbar anesthesia for hip arthroplasty	High dose steroids Surgery on day 4	Marked improvement but no complete recovery

**Table 2:** Risks of rivaroxaban-associated major bleeding events compared to warfarin in selected studies.

	Risk of major bleeding (95% CI)	Risk of gastrointestinal bleeding (95% CI)	Risk of intracranial bleeding (95% CI)
Atrial fibrillation [3]	HR = 1.04 (0.9–1.2)	HR = 1.61 (1.30–1.99)	HR = 0.67 (0.34–0.93)

VTE [8]	HR = 0.49 (0.31–0.79)	RR = 0.56 (0.25–1.27)	RR = 0.10 (0.01–0.78)

## References

[1] Mega J. L. (2011). A new era for anticoagulation in atrial fibrillation. *The New England Journal of Medicine*.

[2] Janssen Pharmaceuticals Inc (2014). *Xarelto Prescribing Information*.

[3] Patel M. R., Mahaffey K. W., Garg J. (2011). Rivaroxaban versus warfarin in nonvalvular atrial fibrillation. *The New England Journal of Medicine*.

[4] Jaeger M., Jeanneret B., Schaeren S. (2012). Spontaneous spinal epidural haematoma during Factor Xa inhibitor treatment (Rivaroxaban). *European Spine Journal*.

[5] Radcliff K. E., Ong A., Parvizi J., Post Z., Orozco F. (2014). Rivaroxaban-induced epidural hematoma and cauda equina syndrome after total knee arthroplasty: a case report. *Orthopaedic Surgery*.

[6] Castillo J. M., Afanador  H. F., Manjarrez E., Morales X. A. (2015). Non-traumatic spontaneous spinal subdural hematoma in a patient with non-valvular atrial fibrillation during treatment with rivaroxaban. *American Journal of Case Reports*.

[7] Dargazanli C., Lonjon N., Gras-Combe G. (2015). Nontraumatic spinal subdural hematoma complicating direct factor Xa inhibitor treatment (rivaroxaban): a challenging management. *European Spine Journal*.

[8] EINSTEIN Investigators, Bauersachs R., Berkowitz S. D. (2010). Oral rivaroxaban for symptomatic venous thromboembolism. *The New England Journal of Medicine*.

[9] Prandoni P. (2012). Anticoagulant treatment of pulmonary embolism: impact and implications of the EINSTEIN PE Study. *European Journal of Haematology*.

[10] Kreppel D., Antoniadis G., Seeling W. (2003). Spinal hematoma: a literature survey with meta-analysis of 613 patients. *Neurosurgical Review*.

[11] Domenicucci M., Ramieri A., Ciappetta P., Delfini R. (1999). Nontraumatic acute spinal subdural hematoma: report of five cases and review of the literature. *Journal of Neurosurgery*.

[12] Hausmann O., Kirsch E., Radü E., Mindermann T. H., Gratzl O. (2001). Coagulopathy induced spinal intradural extramedullary haematoma: report of three cases and review of the literature. *Acta Neurochirurgica*.

[13] Yang N.-R., Kim S. J., Cho Y. J., Cho D. S. (2011). Spontaneous resolution of nontraumatic acute spinal subdural hematoma. *Journal of Korean Neurosurgical Society*.

[14] Bruce-Brand R. A., Colleran G. C., Broderick J. M. (2013). Acute nontraumatic spinal intradural hematoma in a patient on warfarin. *Journal of Emergency Medicine*.

[15] Caputo A. M., Gottfried O. N., Nimjee S. M., Brown C. R., Michael K. W., Richardson W. J. (2013). Spinal epidural hematoma following epidural steroid injection in a patient treated with dabigatran. *JBJS Case Connector*.

[16] Bamps S., Decramer T., Vandenbussche N. (2015). Dabigatran-associated spontaneous acute cervical epidural hematoma. *World Neurosurgery*.

[17] Rader J. P. (1955). Chronic subdural hematoma of the spinal cord: report of a case. *The New England Journal of Medicine*.

[18] Eerenberg E. S., Kamphuisen P. W., Sijpkens M. K., Meijers J. C., Buller H. R., Levi M. (2011). Reversal of rivaroxaban and dabigatran by prothrombin complex concentrate: a randomized, placebo-controlled, crossover study in healthy subjects. *Circulation*.

[19] Levy J. H., Faraoni D., Spring J. L., Douketis J. D., Samama C. M. (2013). Managing new oral anticoagulants in the perioperative and intensive care unit setting. *Anesthesiology*.

[20] Hoffman M., Monroe D. M. (2014). Reversing targeted oral anticoagulants. *Hematology*.

[21] Perzborn E., Heitmeier S., Laux V., Buchmüller A. (2014). Reversal of rivaroxaban-induced anticoagulation with prothrombin complex concentrate, activated prothrombin complex concentrate and recombinant activated factor VII in vitro. *Thrombosis Research*.

[22] Maurice-Szamburski A., Graillon T., Bruder N. (2014). Favorable outcome after a subdural hematoma treated with feiba in a 77-year-old patient treated by rivaroxaban. *Journal of Neurosurgical Anesthesiology*.

[23] Garcia D. A. (2014). The target-specific oral anticoagulants: practical considerations. *Hematology*.

